# Silymarin attenuates cigarette smoke extract-induced inflammation via simultaneous inhibition of autophagy and ERK/p38 MAPK pathway in human bronchial epithelial cells

**DOI:** 10.1038/srep37751

**Published:** 2016-11-22

**Authors:** Diandian Li, Jun Hu, Tao Wang, Xue Zhang, Lian Liu, Hao Wang, Yanqiu Wu, Dan Xu, Fuqiang Wen

**Affiliations:** 1 Division of Pulmonary Diseases, State Key Laboratory of Biotherapy of China and Department of Respiratory and Critical Care Medicine, West China Hospital of Sichuan University, Chengdu, Sichuan, 610041, China

## Abstract

Cigarette smoke (CS) is a major risk of chronic obstructive pulmonary disease (COPD), contributing to airway inflammation. Our previous study revealed that silymarin had an anti-inflammatory effect in CS-exposed mice. In this study, we attempt to further elucidate the molecular mechanisms of silymarin in CS extract (CSE)-induced inflammation using human bronchial epithelial cells. Silymarin significantly suppressed autophagy activation and the activity of ERK/p38 mitogen-activated protein kinase (MAPK) pathway in Beas-2B cells. We also observed that inhibiting the activity of ERK with specific inhibitor U0126 led to reduced autophagic level, while knockdown of autophagic gene Beclin-1 and Atg5 decreased the levels of ERK and p38 phosphorylation. Moreover, silymarin attenuated CSE-induced upregulation of inflammatory cytokines TNF-α, IL-6 and IL-8 which could also be dampened by ERK/p38 MAPK inhibitors and siRNAs for Beclin-1 and Atg5. Finally, we validated decreased levels of both autophagy and inflammatory cytokines (TNF-α and KC) in CS-exposed mice after silymarin treatment. The present research has demonstrated that CSE-induced autophagy in bronchial epithelia, in synergism with ERK MAPK pathway, may initiate and exaggerate airway inflammation. Silymarin could attenuate inflammatory responses through intervening in the crosstalk between autophagy and ERK MAPK pathway, and might be an ideal agent treating inflammatory pulmonary diseases.

Cigarette smoke (CS) contains nearly 5,000 chemicals, most of which are etiological factors in the development of pulmonary diseases, such as chronic obstructive pulmonary disease (COPD). CS exposure has been shown to induce an abnormal inflammatory response in the small airways and alveoli, contributing to airway remodeling and subsequent reduction of the airflow, which is the main characteristic of COPD^1^. It has been demonstrated that bronchial epithelium acts as a main source of overproduction of various cytokines, chemokines and adhesion molecules that modulate other elements of the airway wall and immune cells against CS^2^,^3^. Therefore, mapping the molecular mechanisms for CS-induced alterations in bronchial epithelial cells may offer clues into COPD pathogenesis and treatment.

Macroautophagy (generally referred to as autophagy), a genetically programmed and evolutionarily conserved degradation process^4^, occurs under various cellular stresses, such as starvation, hypoxia and DNA damage^5^. How autophagy shapes the inflammatory response has been discussed in various disease models^6^,^7^,^8^,^9^, and several studies have focused on the functional link between autophagy and inflammation-associated pulmonary pathogenesis, which suggest a critical role of autophagy in inflammation regulation in pulmonary diseases like asthma and acute lung injury (ALI)^10^,^11^. On the other hand, emerging investigations have demonstrated that autophagy can be activated by CS in lung cells^12^,^13^, acting as a deleterious process during COPD pathogenesis such as the apoptosis of lung epithelial cells^13^,^14^. mucin production^15^ and impaired mucociliary clearance^16^,^17^. As abnormal inflammatory response is another well-defined feature of airway epithelium in COPD, we hypothesized that autophagy may also regulate CS-induced inflammation in airway epithelial cells.

Silymarin, a flavonoid compound extracted from the milk thistle (*Silybum marianum*)^18^, is suggested as an anti-inflammatory and antioxidant agent^19^. Our previous study has suggested that pretreatment with silymarin could attenuate CS-induced lung inflammation and oxidative stress in mice, possibly involving ERK/p38 MAPK pathways^20^, which implied a promising role of silymarin in treating CS-induced airway inflammation. Recently, Liu *et al*. have reported that silibin, the main component of silymarin, protected UVB-irradiated L929 cells from apoptotic death by repressing the activation of autophagy^21^. Besides, silibin could inhibit the activation of ERK/p38 MAPK pathways, subsequently decreasing the expression of autophagic genes in an influenza A virus infection model^22^. These evidences indicate that autophagy pathway might be a potential target of silymarin.

In this study, we attempt to elucidate the biological functions of silymarin in CSE-induced inflammation using human bronchial epithelial cell line (Beas-2B). Particularly, we investigated whether silymarin could attenuate CSE-induced inflammatory response via affecting activation of autophagy. Furthermore, we also explored the potential crosstalk between autophagy and ERK/p38 MAPK pathways.

## Material and Methods

### Reagents

Silymarin with a purity of 99% was obtained from Sigma (St Louis, MO, USA) and was dissolved in dimethylsulfoxide (DMSO) ( Sigma, St Louis, MO, USA) to make a stock solution. It was diluted with DMEM with the DMSO concentration kept below 0.1% in cell culture, which had no detectable effects on cells. U0126 and SB203580 were purchased from Cell Signaling Technology (Danvers, MA, USA) and Selleck Chemicals (Houston, TX, USA), respectively. Primary antibodies against LC3B, phospho-ERK, phospho-p38, β-actin and horseradish peroxidase-conjugated second antibodies were all pruchased from Cell Signaling Technology. The SuperSignal West Pico Chemiluminescent Substrate for horseradish peroxidase enzyme was obtained from Pierce (Rockford, IL, USA).

### Preparation of CSE

CSE was prepared as previously described^23^. Briefly, mainstream smoke from 15 cigarettes (Jiao Zi, Chengdu Cigarette Factory, Chengdu, China; 1.1 mg nicotine and 11 mg tar per cigarette) was drawn slowly into a 50-ml syringe and bubbled through 50 ml of DMEM pre-warmed in a water bath at 37 °C. The preparation, considered to be 100% CSE, was titrated to pH 7.4 and sterilized with a 0.22 μm filter (Millipore, Bedford, MA, USA), and then stored at −80 °C. Serum-free cell culture medium was used to dilute 100% CSE to the required CSE concentrations.

### Cell culture

Beas-2B cells obtained from the American Type Culture Collections (Manassas, VA, USA) were cultured in Dulbecco’s Modified Eagle Medium (DMEM) supplemented with 10% fetal bovine serum (FBS), 50 U/ml penicillin G sodium and 50 μg/ml streptomycin sulfate (Invitrogen, Carlsbad, CA, USA) at 37 °C in 5% CO_2_ -enriched air. All experiments were performed on logarithmically growing cells. Cell layers were 70–80% confluent at the time of CSE exposure.

### Cell viability assay

Cells were seeded at an initial density of 5000 cells per well onto 96-well plates and allowed to attach overnight, before treated with indicated concentrations of CSE or silymarin or in combination (cells were pretreated with silymarin 1 h before CSE exposure). After 24 h, 10 μl reagent of Cell Counting Kit-8 (CCK8, Dojindo Laboratories, Tokyo, Japan) was added to each well and incubated for 2 h. Absorbance values were recorded using a Model 680 microplate reader (Bio-Rad, Hercules, CA, USA) at an absorbance of 450 nm.

### Transient transfection of siRNA

Beas-2B cells were cultured in 12-well plate overnight to 40–60% confluence in complete medium without antibiotics. Validated small-interfering RNA (siRNA) oligonucleotides specific to Atg5, Beclin-1 and negative control siRNA were purchased from Sigma (St Louis, MO, USA). Transient transfections were performed using HiPerFect Transfection Reagent (Qiagen,Valencia, CA, USA) according to the manufacturer’s protocols. Briefly, cells were incubated with siRNA-HiPerFect complexes for 16 h. The medium was then removed and replaced with fresh complete medium. After transfection for 24 h, cells were exposed to CSE. The control and CSE groups received the same volume transfection reagents in parallel to avoid stress-induced secretion of cytokines in response to transfection.

### Fluoresecence microscope imaging of autophagy after pEGFP-LC3 transfection

The pEGFP-LC3 plasmid expressing the autophagosome marker protein microtubule-associated protein 1 light chain 3 beta (LC3) (Addgene plasmid 21073) was kindly provided by Tamotsu Yoshimori, Department of Genetics, Osaka University Graduate School of Medicine, Japan^24^. Cells were transfected with the plasmid by use of Attractene (Qiagen, Valencia, CA, USA) following the manufacturer’s instructions and were maintained on coverslips in 24-well plates. After transfection for 24 h, cells were treated with indicated concentrations of CSE or silymarin or in combination (cells were pretreated with silymarin 1 h before CSE exposure). DAPI (Sigma, St Louis, MO, USA) was used to stain the nuclei. The images of cells were acquired using a Nikon fluorescence microscope. Autophagy was detected by monitoring the formation of fluorescent puncta of autophagosomes in pEGFP-LC3 transfected cells.

### Treatment of Animals

Animals were treated as previously described^20^. Briefly, specific pathogen-free male BALB/c mice (6–8 weeks) were randomly divided into four groups (n = 6 per group): control group (C), which received placebo and was not exposed to CS; CS-exposed group (CS), which received placebo and was exposed to CS; CS-exposed low-dose silymarin group (CS+ SiL), which received 25 mg/kg silymarin (i.p., q. d.) and was subsequently exposed to CS; and CS-exposed high- dose silymarin group (CS+SiH), which received 50 mg/kg silymarin (i.p., q. d.) and was subsequently exposed to CS. For cigarette smoke exposure experiment, animals were exposed to mainstream CS at 250 mg total particulate matter (TPM)/m^3^ for 4 h per day, 6 days per week for 4 weeks using a Baumgartner-Jaeger CSM2082i automated cigarette smoke generator (CH Technologies, Westwood, NJ, USA). A commercially available cigarette was employed (Jiaozi, China Tobacco Chuanyu Industrial Co., Ltd.; 1.1 mg nicotine and 11 mg tar per cigarette). In parallel, C group were exposed to air following the same schedule. After CS exposure for 4 weeks, the mice in all groups were sacrificed with intraperitoneal 3% sodium pentobarbital, followed by exsanguination from the right ventricle to allow tissue sample collection. All experimental protocols involving the mice were in accordance with the Guide for the Care and Use of Laboratory Animals.The study protocol was approved by the Animal Care and Use Committee of West China Hospital.

### Collection of Bronchoalveolar Lavage Fluid (BALF)

The right lung was lavaged three times with 0.5 ml of sterile saline, with a recovery rate of 95%. The lavage fluid was centrifuged at 1,000 g for 5 min, and the supernatant was stored at −80 °C for analysis of cytokines using enzyme-linked immunosorbent assay (ELISA).

### Western Blot analysis

Cells or lung tissues **w**ere lysed in RIPA buffer containing 50 mM Tris–HCl (pH 7.4), 150 mM NaCl, 1% NP-40, 0.5% sodium deoxycholate, 2 mM sodium fluoride, 2 mM EDTA, 0.1% SDS, and PMSF. Protein concentrations were determined by a BCA protein assay kit (Thermo Fisher Scientific Inc., MA, USA). Total protein (20 μg) was fractionated by 10% SDS polyacrylamide gel electrophoresis and transferred to PVDF membranes. Membranes were blocked for 1 h at room temperature with 5% BSA in TBS-Tween and incubated overnight at 4 °C with the appropriate primary antibodies. After incubation with horseradish peroxidase-conjugated second antibodies, the immune complexes were detected with SuperSignal West Pico chemiluminescent substrate. Band intensities were quantified using computerized image analysis (QuantityOne-software, Bio-Rad, Hercules, CA, USA).

### Real-Time PCR

The total RNA was isolated from Beas-2B cells using the Total RNA Kit I (Omega Bio-tek, Norcross, GA, USA) according to the manufacturer’s protocol. cDNA was synthesized from using the iScript cDNA Synthesis Kit (Bio-Rad, Hercules, CA, USA). Quantitative real-time RT-PCR analysis was conducted in triplicates by the CFX96 real-time PCR detection system using SsoFast EvaGreen Supermix according to the manufacturer’s specifications (Bio-Rad, Hercules, CA, USA). The primer sequences were as follows: Atg5 forward 5′-TAGTATCATCCCACAGCCAACAG-3′, reverse 5′-AGTAAGACCAGCCCAGTTGCC-3′; Beclin-1 forward 5′-GCTGAGGGATGGAAGGGTCT-3′, reverse 5′-CGCCTGGGCTGTGGTAAGT-3′; β-actin forward 5′-CTGCCAAGTGGGTGGTATAGAG-3′, reverse 5′-GGTAGTCCATAGTGAAGGCGAAC-3′. The efficiency of primers for Atg5, Beclin-1 and β-actin were 102%, 89.5% and 114%, respectively. All data were normalized to β-actin gene expression, and relative expression levels were determined using the 2 −ΔΔCt method.

### Enzyme-linked immunosorbent assays (ELISA)

Levels of cytokine release (TNF-α, IL-6 and IL-8 in culture supernatants; TNF-α, IL-6 and KC in BALF) were determined by ELISA kit (ExCell Biology, Shanghai, China) according to the manufacturer’s protocol. The absorbance of 570 nm was measured by a Model 680 microplate reader (Bio-Rad, Hercules, CA, USA).

### Statistical analysis

All values were expressed as mean ± SEM. Differences among multiple groups were analyzed by one-way analysis of variance (ANOVA). Significance was defined by a p value < 0.05 (two-tailed). All statistical calculations were carried out in SPSS (version 22.0, SPSS Inc., USA).

## Results

### Evaluation of cell viability following exposure to CSE and silymarin

**Figure 1:**
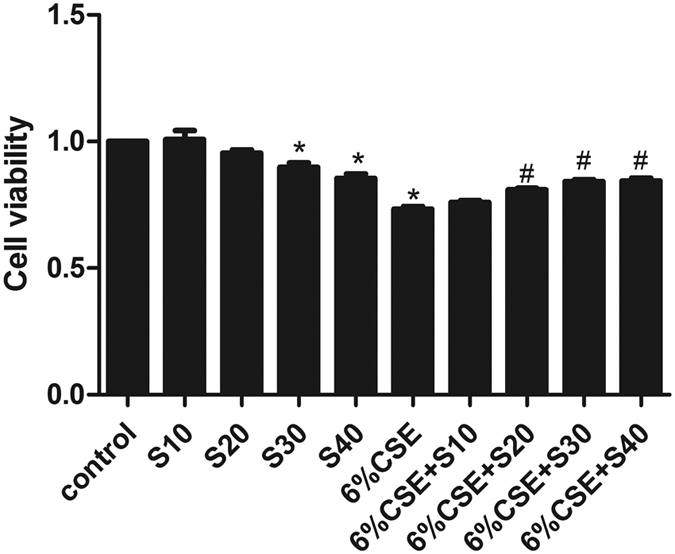
Effect of silymarin on cell viability in CSE-exposed Beas-2B, as determined by CCK8 assay. Results are representative of three independent experiments. Values are expressed as mean ± SEM (n = 3). *P < 0.05 with respect to the control group; ^#^*P* < 0.05 with respect to the CSE-exposed group. S, silymarin.

Cells were exposed to various concentrations of CSE (0% to 10%) for 24 h, after which cell growth was determined by CCK8 assay. The results showed that CSE inhibited the growth of cells in a dose-dependent manner and exerted cytotoxic effect when the concentration exceeded 6% (Supplementary Fig. S1). Next, we treated Beas-2B cells with 6% CSE or silymarin (10, 20, 30 or 40 μM) or in combination for 24 h. The results revealed that the viability of Beas-2B cells was dose-dependently improved by silymarin treatment (Fig. 1). However, due to the obvious growth inhibitory effect of silymarin at concentrations higher than 30 μM, silymarin at a concentration of 20 μM was selected for further study (Fig. 1).

### Autophagy was activated by CSE and reversed by silymarin in Beas-2B cells

**Figure 2:**
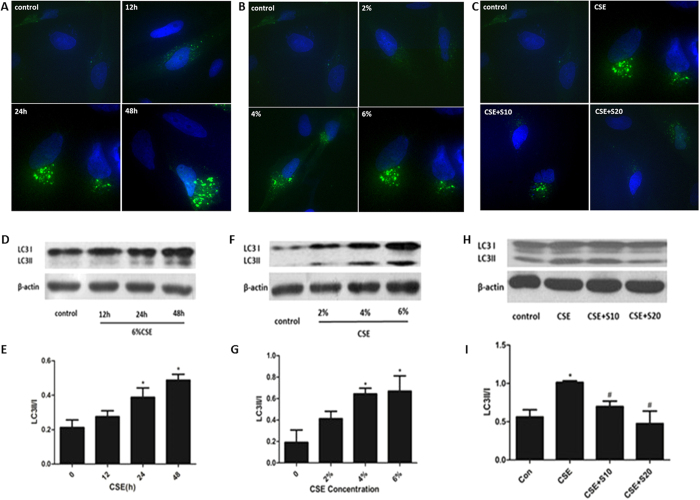
Autophagy activation in response to CSE and silymarin treatment. Beas-2B cells were treated with 6%CSE for different time period, indicated concentrations of CSE for 24 h or both 6%CSE and silymarin (10 μM, 20 μM) for 24 h. Formation of pEGFP-LC3 puncta in Beas-2B cells was analyzed by immunofluorescence under fluorescence microscopy (×400) (**A**–**C**). Expressions of LC3I and LC3II were measured by Western Blot (**D**,**F**,**H**). Densitometry was performed and the ratio of LC3II/I were calculated (**E**,**G**,**I**). Results are representative of three independent experiments. Values are expressed as mean ± SEM (n = 3). *P < 0.05 with respect to the control group; ^#^*P* < 0.05 with respect to the CSE-exposed group. S, silymarin.

Autophagy can be activated in cells in response towards various stresses, such as oxidative stress, hypoxia, nutrient deprivation, DNA damage and intracellular pathogens^25^,^26^,^27^. In the present study, CSE activated the autophagic process in time-and dose-dependent manner, as shown by the marked increase of pEGFP-LC3 puncta in Beas-2B cells (Fig. 2A,B) and accelerated conversion of LC3-I to LC3-II in the western-blot analysis (Fig. 2D–G). However, pretreatment with silymarin (10 μM or 20 μM) attenuated this induction of autophagy (Fig. 2C,H,I) without changing the basal level of autophagy (Supplementary Fig. S2).

### Silymarin suppressed the activity of ERK/p38 MAPK pathway in CSE-treated Beas-2B cells

**Figure 3:**
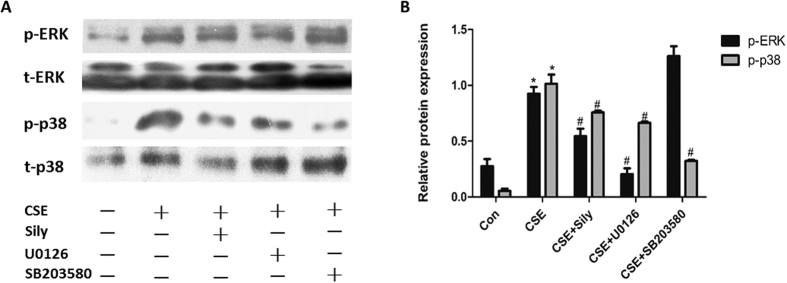
Effect of silymarin on ERK/p38 MAPK pathway in CSE-exposed Beas-2B cells. Cells were pretreated with silymarin (20 μM), U0126 (10 μM) or SB203580 (10 μM) before exposed to 6%CSE for 24 h. (**A**) Phosphorylated and total levels of ERK and p38 were measured by Western Blot. (**B**) Densitometry was performed and the ratio of p-p38/t-p38 and p-ERK/t-ERK were calculated. Results are representative of three independent experiments. Values are expressed as mean ± SEM (n = 3). *P < 0.05 with respect to the control group; ^#^*P* < 0.05 with respect to the CSE-exposed group.

We next investigated whether silymarin possessed the ability to inhibit ERK/p38 MAPK pathway in CSE-treated Beas-2B cells. The phosphorylation levels of ERK and p38 were measured by Western-blot. As shown in Fig. 3, both ERK and p38 phosphorylation levels were enhanced in the CSE-treated cells, while pretreatment with silymarin effectively prevented the CSE-induced upregulation of p-ERK and p-p38, compared with the inhibitors of ERK/p38 MAPK pathway, U0126 and SB203580. However, silymarin did not change the basal levels of ERK and p38 phosphorylation (Supplementary Fig. S3).

### ERK MAPK pathway regulated autophagy activation in CSE-treated Beas-2B cells

**Figure 4:**
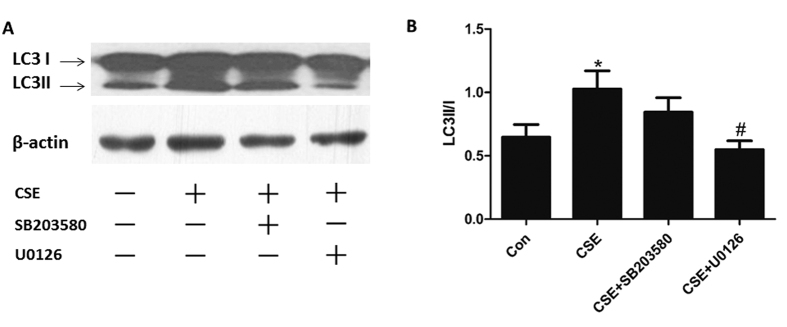
ERK MAPK pathway regulated autophagy activation in CSE-treated Beas-2B cells. Cells were pretreated with U0126 (10 μM) or SB203580 (10 μM) before exposed to 6%CSE for 24 h. (**A**) Expressions of LC3I and LC3II were measured by Western Blot. (**B**) Densitometry was performed and the ratio of LC3II/I were calculated. Results are representative of three independent experiments. Values are expressed as mean ± SEM (n = 3). *P < 0.05 with respect to the control group; ^#^*P* < 0.05 with respect to the CSE-exposed group.

Some reports have shown that autophagy could be regulated by ERK and p38 MAPK pathways under different conditions^28^,^29^,^30^. In this study, suppressing the activity of ERK signaling pathway with its specific inhibitor U0126 led to reduced autophagic level, as reflected by the blocking of LC3 conversion in CSE-treated Beas-2B cells. However, p38 MAPK inhibitor SB203580 showed no significant influence on the ratio of LC3II to LC3I in this model (Fig. 4A,B).

### Blocking autophagy suppressed CSE-induced ERK and p38 phosphorylation in Beas-2B cells

**Figure 5:**
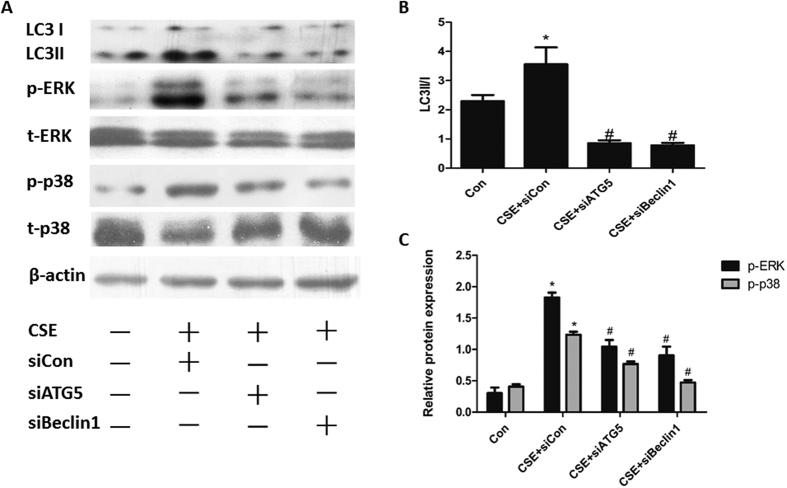
Blocking autophagy suppressed CSE-induced ERK and p38 phosphorylation in Beas-2B cells. Cells were transfected with Atg5 siRNA (100 nM), Beclin-1 siRNA (100 nM) or negative control siRNA before exposed to 6%CSE for 24 h. (**A**) Expressions of LC3I and LC3II, phosphorylated and total levels of ERK and p38 were measured by Western Blot. Densitometry was performed and (**B**) the ratio of LC3II/I, (**C**) p-p38/t-p38 and p-ERK/t-ERK were calculated. Results are representative of three independent experiments. Values are expressed as mean ± SEM (n = 3). *P < 0.05 with respect to the control group; ^#^*P* < 0.05 with respect to the CSE-exposed group.

To explore the potential interaction between autophagy and ERK/p38 MAPK pathways in Beas-2B cells, knockdown of the autophagic gene Beclin-1 and Atg5 by siRNA were used to repress the induction of autophagic flux. Transfection of Beclin-1 and Atg5 siRNA, efficiently and specifically reduced Beclin-1 and Atg5 mRNA expression, respectively (Supplementary Fig. S4). Further, treatment with Beclin-1 and Atg5 siRNA at 100 nM before 6% CSE stimulation reduced the conversion of LC3 proteins (Fig. 5A,B). By selective knockdown of these autophagy related genes, we observed decreased levels of ERK and p38 phosphorylation (Fig. 5A,C) when CSE presented, suggesting that blocking autophagy might inhibit the ERK and p38 axis.

### Silymarin attenuated CSE-stimulated inflammatory cytokine release in an autophagy- and ERK/p38 MAPK-dependent manner

**Figure 6:**
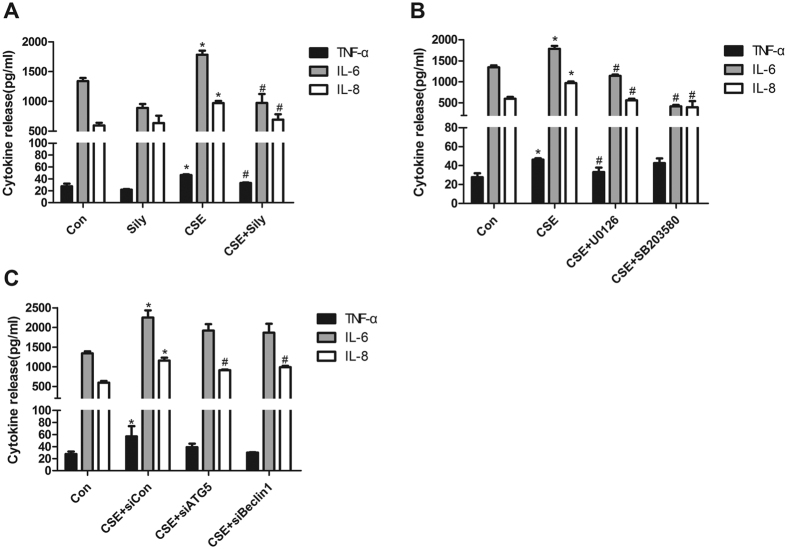
Silymarin attenuated CSE-stimulated inflammatory cytokine release in an autophagy- and ERK/p38 MAPK-dependent manner. (**A**) Beas-2B cells pretreated with silymarin (20 μM), (**B**) ERK/p38 MAPK inhibitors U0126(10 μM) and SB203580(10 μM), or (**C**) negative control/Atg5/Beclin-1 siRNA (100 nM) were exposed to 6%CSE for 24 h. The secretion of TNF-α, IL-6 and IL-8 in supernatants were detected by ELISA. Data were averaged from a duplicate of each sample and from three independent experiments. Values are expressed as mean ± SEM (n = 3). *P < 0.05 with respect to the control group; ^#^*P* < 0.05 with respect to the CSE-exposed group.

ELISA for TNF-α, IL-6 and IL-8 in the culture supernatant was used to determine the effect of silymarin on CSE-induced pro-inflammatory response. As shown in Fig. 6A, silymarin pretreatment significantly reduced the CSE-induced increases of TNF-α, IL-6 and IL-8 release in the supernatants.

To further investigate whether the anti-inflammatory effects of silymarin were partly mediated by autophagy or ERK/p38 MAPK pathways, selective knockdown of autophagy related genes (Atg5 and Beclin-1) were utilized to inhibit autophagy flux, while selective inhibitors for ERK (U0126) and p38 (SB203580) were also used before CSE treatment. The results indicated that inhibiting ERK/p38 MAPK could decrease the CSE-induced release of IL-6 and IL-8 (Fig. 6B), whereas blocking autophagy significantly decreased the production of proinflammatory cytokine IL-8 (Fig. 6C). Combined with findings of previous parts of this study, these results suggested that ERK/p38 MAPK and autophagy signaling may regulate CSE-induced inflammatory cytokine release, while inhibitory effect of silymarin on these pathways could attenuate such CS-related airway inflammation.

### Silymarin inhibited autophagy in a mouse model of airway inflammation

**Figure 7:**
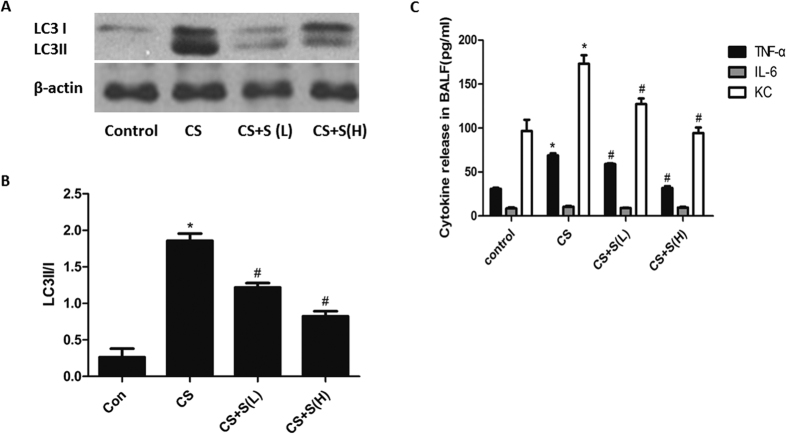
Effect of silymarin on autophagy activation and inflammatory cytokine release in mice lungs. (**A**) Expressions of LC3I and LC3II were measured by Western Blot. (**B**) Densitometry was performed and the ratio of LC3II/I were calculated. (**C**) The secretion of TNF-α, IL-6 and KC in BALF were detected by ELISA. Results are representative of three independent experiments. Values are expressed as mean ± SEM (n = 3). *P < 0.05 with respect to the control group; ^#^*P* < 0.05 with respect to the CSE-exposed group.

Results from our previous work have implied that silymarin could decrease the activity of ERK/p38 MAPK pathway in CS-treated mice^20^. Using this model, we further validated whether silymarin influenced activation of autophagy and regulated CS-induced airway inflammation. The results demonstrated that the LC3II/I in lung tissues of mice were increased after CS exposure for 4 weeks, which could be reverted by silymarin treatment in a dose-dependent manner (Fig. 7A,B). Moreover, CS-induced elevation of KC in BALF, along with TNF-α, could be decreased by silymarin pretreatment (Fig. 7C).

## Discussion

Silymarin administration has been used for various inflammatory conditions. The therapeutic potential of silymarin in several pulmonary disease models has recently attracted attention. Silymarin reduced sepsis-induced oxidative damage in lung tissues and inhibited the release of inflammatory mediators^31^. Silibinin, the main component of silymarin, can also protect against OVA-induced airway inflammation by downregulating pro-inflammatory and Th2 cytokines^32^. A more recent paper reported that silymarin was able to increase ciliary beat frequency and mucociliary clearance *in vivo*^33^. Consistent with our previous study in CS-exposed mice which showed that silymarin could dampen airway inflammatory responses and oxidative stress, the present study further demonstrated its anti-inflammatory effect and the possible underlying mechanisms in CSE-induced Beas-2B cells, which may be more related to human COPD as bronchial epithelial cells play a crutial role in disease pathogenesis.

Autophagy is initiated by induction of several autophagy genes including those that express microtubule-associated protein light chain 3 (LC3), Beclin-1, and other autophagy-related proteins, playing a critical role in the maintenance of intracellular homeostasis under both physiological and pathological conditions^34^,^35^,^36^. Increasing evidence has suggested cross-talk between autophagy and apoptosis in lung epithelial cells subjected to CSE treatment^37^. Down-regulation of the activated autophagic and apoptotic pathways controls lung cell death and the emphysema development in mice exposed to CS^13^,^14^,^38^. Although autophagy is recently emerging as an important process for the regulation of innate immunity and inflammation, the role of autophagy in the mechanisms of lung inflammation has been controversial with studies that have suggested both protective and injurious aspects. Rescue of dysfunctional autophagy has been shown to play a protective role against hyperinflammatory responses from cystic fibrosis cells^39^, while autophagy deficiency in alveolar macrophages resulted in greater silica-induced inflammation^40^. In contrast, inhibition of autophagy in lung macrophages abolished mechanical ventilation-induced nucleotide-binding oligomerization domain-like receptor containing pyrin domain 3 (NLRP3) inflammasome activation and lung inflammatory injury^41^. In avian influenza A-infected lung epithelial cells, blocking autophagy not only reduced the cell death^42^,^43^, but also decreased the production of proinflammatory cytokines^43^. Besides, genetic blockage of autophagy could markedly reduce PM2.5-induced airway inflammation and mucus hyperproduction^44^. Whether autophagy is protective or detrimental in lung inflammation seems to depend on the extent of its activation, specific stimuli/circumstances, and specific cell type. Consistent with the latter studies, our data implied that autophagy was deleterious in CSE-induced inflammation in bronchial epithelia, as autophagy inhibition resulted in attenuated proinflammatory cytokine release. These findings were further supported by a recent study showing that inhibition of autophagy by 14, 15-epoxyeicosatrienoic acid (EET) suppressed CS-induced inflammation in Beas-2B cells^45^. The anti-inflammatory effect of inhibiting autophagy may be exerted by the subsequent accumulation of p62 and ubiquitinated proteins^46^, which then promotes translocation of Nrf2 into the nucleus^47^ and regulates antioxidant defenses in inflammatory lung diseases. Thus, targeting autophagy signaling (i.e. silymarin administration or 14, 15-EET) in airway epithelial cells might be effective for CS-induced airway inflammation and exacerbation of various chronic lung diseases.

MAPKs, including p38, ERK and c-Jun N-terminal kinase (JNK), are members of a ubiquitous protein serine/threonine kinase family responsible for signal transduction in eukaryotic organisms^48^,^49^,^50^. It is well established that MAPK activation is implicated in the production of many inflammatory mediators present in the COPD lung or CSE-induced airway epithelial cells^51^,^52^,^53^. Some reports have demonstrated that MAPK pathway could regulate the level of autophagy. Addition of the MEK/ERK inhibitor U0126, blocked the dissociation of Bcl-2–Beclin1 in the setting of enhanced autophagy, suggesting that ERK1/2-mediated phosphorylation of Bcl-2 regulated starvation-induced autophagy^30^. ERK1/2 can be also suppressed by amino acids to inhibit autophagy^29^. Moreover, p38 MAPK inhibitor was shown to directly abrogate palmitate-induced conversion of LC3I to LC3II^28^. Our data revealed that suppression of ERK phosphorylation reduced autophagy in CSE-treated Beas-2B cells, implying a positively regulating role of ERK MAPK in CSE-induced autophagy. Meanwhile, we found that inhibition of autophagy led to a decreased level of ERK phosphorylation, suggesting that CSE-induced inflammatory response in Beas-2B cells might be induced and strengthened by a positive feedback loop of ERK MAPK pathway and autophagy activation. Although the complex interactions exist between ERK MAPK and autophagy, it is speculated that ERK-mTOR signaling may play a role in regulating autophagy. Most of autophagy induction conditions such as low cellular energy levels have been shown to inhibit mTOR activity, suggesting a tight, inverse coupling of autophagy induction and mTOR activation^54^. Wang *et al*. reported that transiently or moderately activated MEK/ERK inhibited mTOR activity, so as to cause moderately enhanced Beclin-1 and cytoprotective autophagy, while sustained MEK/ERK activation caused cytodestructive autophagy^55^. However, reports concerning the direct effect of autophagy on the activation of ERK are limited. One recent study by Martinez-Lopez *et al*. showed that deleting Atg7 or Atg5 or blocking LC3 lipidation or Atg5-Atg12 conjugation decreased ERK phosphorylation^56^, indicating that the cellular availability of autophagic structures could determine the degree of ERK phosphorylation. In their study, it was speculated that LC3-II-positive membranes and Atg5-Atg12-positive preautophagosomes might serve as cellular signaling platforms which would facilitate ERK phosphorylation by promoting coordination of the Raf-MEK-ERK cascade. Therefore, interfering this loop at the same time might achieve a better prevention against CS-induced airway inflammation.

In summary, the present research has demonstrated that CSE-induced autophagy activation in bronchial epithelia, acting in synergism with ERK MAPK pathway, may initiate and exaggerate airway inflammation. Silymarin could attenuate inflammatory responses, at least in part, via suppressing ERK/p38 MAPK pathway activity and autophagy. Thus, therapeutic improvements in CS-associated airway inflammation might be facilitated through intervening in the crosstalk between autophagy and ERK MAPK pathway, and silymarin is likely to be an ideal agent for use in inflammatory pulmonary diseases.

## Additional Information

**How to cite this article**: Li, D. *et al*. Silymarin attenuates cigarette smoke extract-induced inflammation via simultaneous inhibition of autophagy and ERK/p38 MAPK pathway in human bronchial epithelial cells. *Sci. Rep.* **6**, 37751; doi: 10.1038/srep37751 (2016).

**Publisher’s note:** Springer Nature remains neutral with regard to jurisdictional claims in published maps and institutional affiliations.

## Supplementary Material

Supplementary Information
