# Supplementary material for: Silymarin attenuates cigarette smoke extract-induced inflammation via simultaneous inhibition of autophagy and ERK/p38 MAPK pathway in human bronchial epithelial cells
Source: Sci Rep. 2016 Nov 22;6:37751. doi: 10.1038/srep37751 (PMC5118787; doi:10.1038/srep37751)
Supplement: Supplementary Information [file srep37751-s1.pdf]

Silymarin attenuates cigarette smoke extract-induced inflammation via simultaneous inhibition of autophagy and ERK/p38 MAPK pathway in human bronchial epithelial cells

Diandian Li, Jun Hu, Tao Wang, Xue Zhang, Lian Liu , Hao Wang, Yanqiu Wu, Dan Xu and Wen Fuqiang

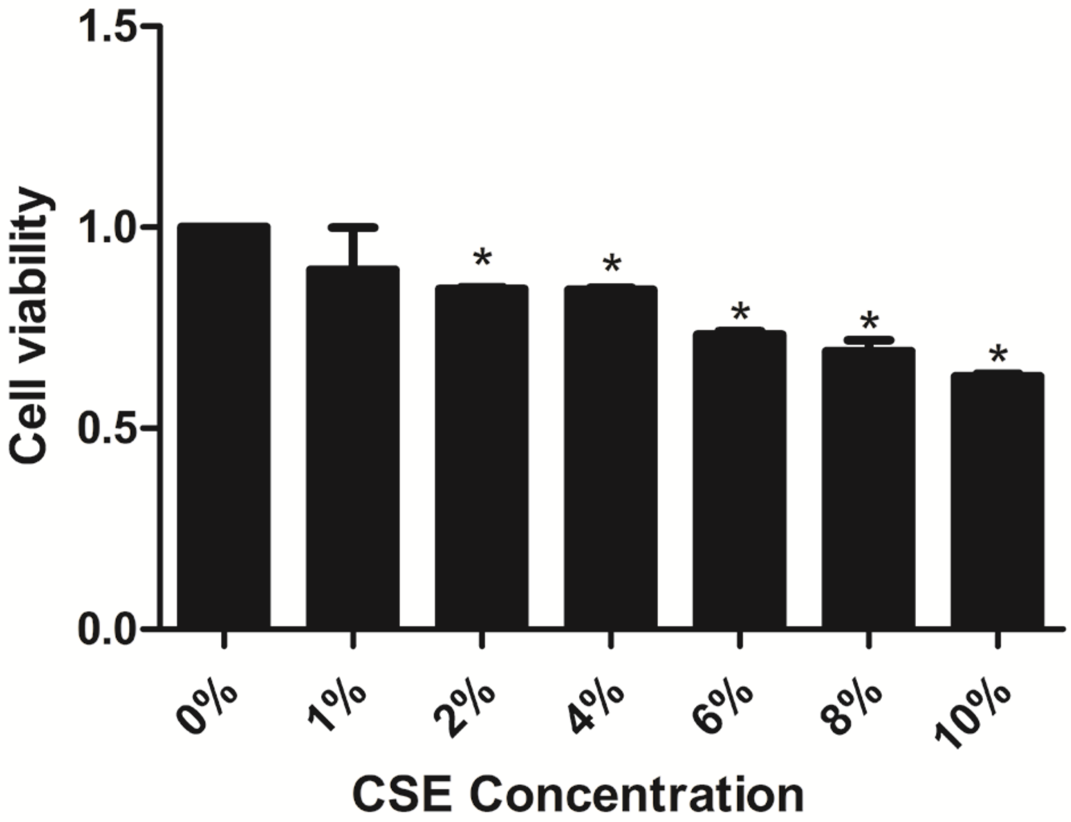

Fig.S1 CSE inhibited cell growth in a dose-dependent manner, as determined by the CCK8 assay. Results are representative of three independent experiments. Values are expressed as mean±SEM (n=3). *, $P < 0.05$ with respect to the control group.

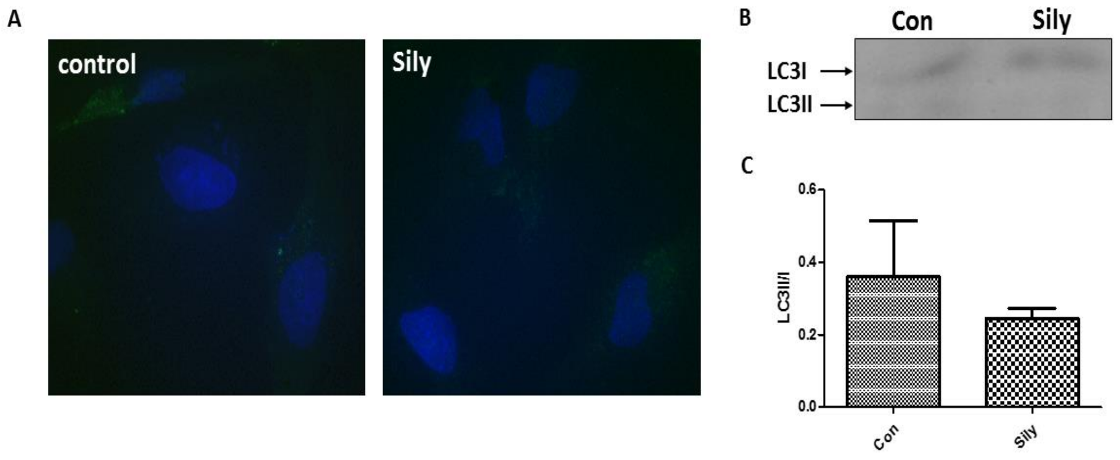

Fig.S2 Autophagy activation in response to silymarin treatment. Beas-2B cells were treated with silymarin (20μM) for 24h. Formation of pEGFP-LC3 puncta in Beas-2B cells was analyzed by immunofluorescence under fluorescence microscopy (×400) (A). Expressions of LC3I and LC3II were measured by Western Blot (B). Densitometry was performed and the ratio of LC3II/I were calculated (C). Results are representative of three independent experiments. Values are expressed as mean±SEM (n=3).

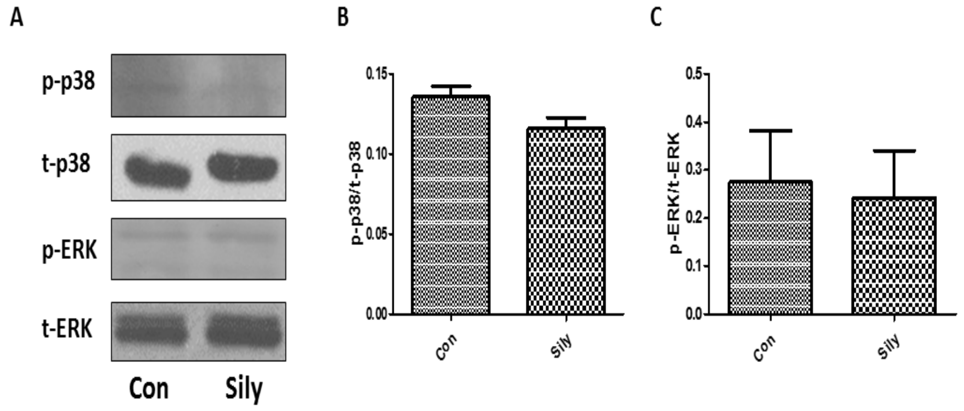

Fig.S3 Effect of silymarin on ERK/p38 MAPK pathway in Beas-2B cells. Cells were pretreated with silymarin (20μM) for 24h. Phosphorylated and total levels of ERK and p38 were measured by Western Blot (A). Densitometry was performed and the ratio of p-p38 /t-p38 and p-ERK /t-ERK were calculated (B−C). Results are representative of three independent experiments. Values are expressed as mean±SEM (n=3).

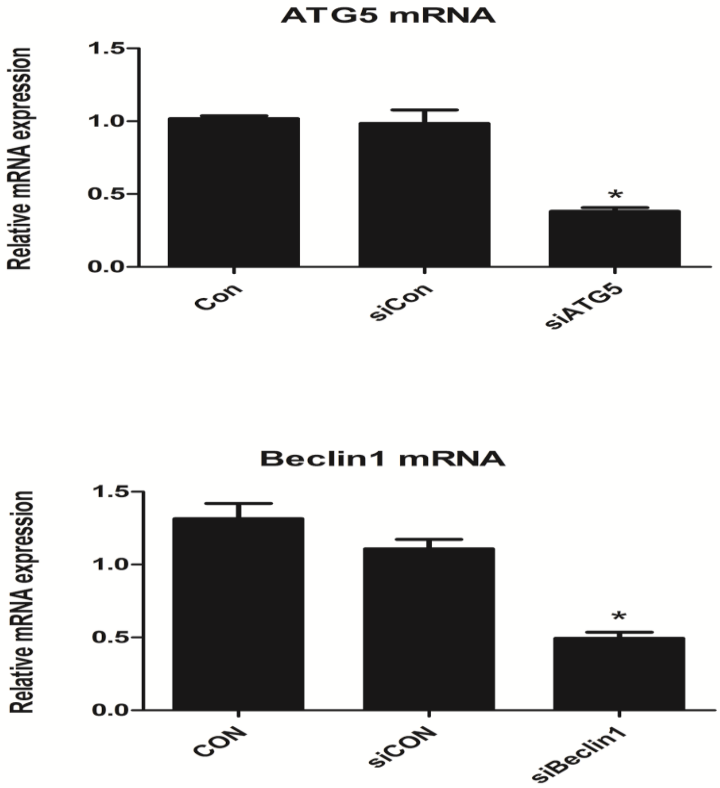

Fig.S4 Transfection of Atg5 and Beclin-1 siRNA, efficiently and specifically reduced Atg5 and Beclin-1 mRNA expression, respectively. The mRNA expressions of Atg5 and Beclin-1 were assayed by real-time RT-PCR. The levels of mRNA were normalized to the β-actin values. Results are representative of three independent experiments. Values are expressed as mean±SEM (n=3). *, $P<0.05$ with respect to the control group.
